# ﻿*Agrotisvillenensis*—a new species of Noctuinae (Lepidoptera, Noctuidae) from the southeastern Iberian Peninsula

**DOI:** 10.3897/zookeys.1239.147164

**Published:** 2025-05-20

**Authors:** José Luis Yela, David Molina, Antonio S. Ortiz

**Affiliations:** 1 Grupo DITEG, Área de Zoología, Facultad de Ciencias Ambientales and ICAM, Universidad de Castilla-La Mancha, Avda, Carlos III, s.n.; Campus Real Fábrica de Armas, E-45071 Toledo, Spain Universidad de Castilla-La Mancha, Avda Toledo Spain; 2 c/ Félix Rodríguez de la Fuente 1, 1º, p 6, 03400, Villena (Alicante), Spain Unaffiliated Villena Spain; 3 Department of Zoology and Physical Anthropology, University of Murcia, Campus de Espinardo, E-30100 Murcia, Spain University of Murcia Murcia Spain

**Keywords:** DNA barcode, integrative taxonomy, new species

## Abstract

*Agrotisvillenensis***sp. nov.** is described from the Iberian Peninsula. Differential superficial, genital and genetic (barcode) characters from its closest Iberian and European relative species, *Agrotisvestigialis* (Hufnagel, 1766), are presented. Morphologically, the new species is best characterized in the male genitalia by the shape of the basal vesica and the presence of a median diverticulum and in the female genitalia by its comparatively long appendix bursae. The barcode of *A.villenensis* differs from those of related species and is assigned a unique BIN.

## ﻿Introduction

The family Noctuidae Latreille, 1809 includes approximately 11,772 species and 1,089 genera worldwide (Nieukerken et al. 2011). The subfamily Noctuinae Latreille, 1809 includes about 300 species in the nearly worldwide genus *Agrotis* Ochsengeimer, 1816 (Poole 1989; San Blas 2014). Eighty *Agrotis* species occur in the Palaearctic region (Poole 1989). Fibiger (1990) listed 32 species in Europe; Fibiger et al. (2010) increased the number to 41 after *Powellinia* Oberthür, 1912 was synonymized with *Agrotis* (Lafontaine 2004). At present, 50 species are known to occur in the western Palaearctic (Lepiforum 2024) when the fauna of the Canary Islands is included.

Although species groups based in external similarity have been proposed (Fibiger 1990; Lafontaine 2004), many species in the genus are closely similar. Even more, their genitalia, both external and internal, are confusingly alike in many species (Fibiger 1997; San Blas 2014). The most significant species differences are usually in the male antennae (Curtis 1827; Fibiger 1990), which are typically bipectinate with biciliate lamellae bearing double tufts of more or less long cilia (Lafontaine 2004). The male genitalia are characterized by a narrow valva, with a corona of numerous long spiniform setae, a thick short clasper arising from the centre of the valve and directed posterodorsad, and a well-differentiated rounded pouch on the costal margin of the valva at the base of the clasper; a broad juxta, with a short pointed tip directed towards saccus; a phallus with a narrow, ventral, hooklike sclerotized band continued in a narrow sclerotized spiny or scobinate bar at the left tip with the spines directed away from the phallus, and a vesica with a basal swelling, usually with a lateral conical-triangular diverticulum, followed by a coil into a narrow, long looping tube, often with a long bulbous distal swelling (Fibiger 1997; Lafontaine 2004; San Blas 2014). The female genitalia have a membranous, stretchy ductus bursae and bisaccate corpus bursae with curved corpus main segment and a longer looping appendix bearing the ductus seminalis at the apex; and long, slender apophyses (Fibiger 1997; Lafontaine 2004).

During recent studies of the Noctuoidea of the Iberian Peninsula, the authors found a new *Agrotis* species of the *vestigialis* group that has significant morphological differences from all other Iberian *Agrotis* species and a unique barcode. The aim of the present study is to describe this species as *Agrotisvillenensis* sp. nov., comparing it with its putative sister species, *A.vestigialis*.

## ﻿Material and methods

### ﻿Morphological study

This study was based on the morphology of 11 adult moths collected (*n* = 7) or photographed (*n* = 4) in Peña Rubia, Villena, in the Alicante province of southwest Spain.

**Figure 1.**
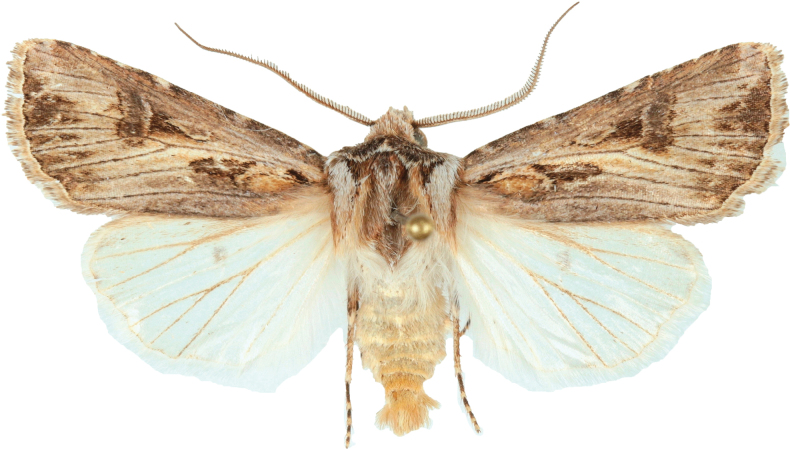
Holotype of *Agrotisvillenensis* sp. nov., from Peña Rubia, Villena, Alicante, Spain. Photograph: Mateo Yela Berzosa.

**Figure 2.**
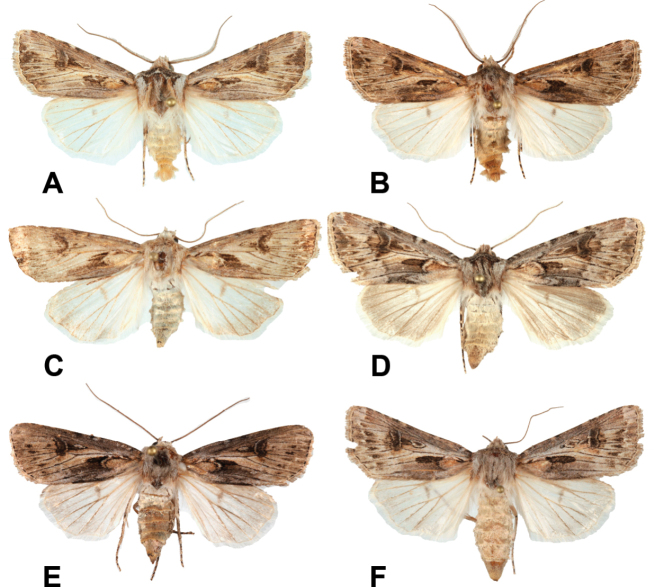
External variability of *Agrotisvillenensis* sp. nov. **A** holotype **B–F** paratypes. Photograph: Mateo Yela Berzosa.

**Figure 3.**
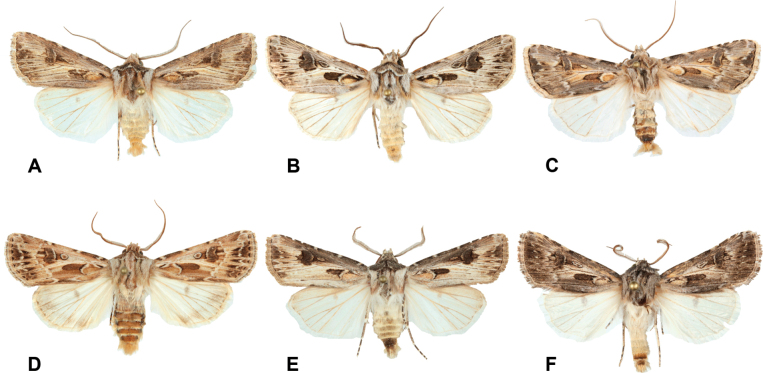
Male specimens of the Iberian nominal species of the *Agrotisvestigialis* species group, for comparison **A***A.villenensis* sp. nov., holotype **B***Agrotisvestigialis*, Playa La Rubina, Empuriabrava, Gerona **C***Agrotisyelai*, Castronuño, Valladolid **D***Agrotischaroae*, Playa de Oyambre, San Vicente de la Barquera, Cantabria **E***Agrotissabulosa*, Huelva **F***Agrotisgarretasorum*, Toril, Cáceres. Photographs: J. L. Yela.

They were examined externally to assess differences in their color and wing pattern based on the pertinent taxonomic traits of *Agrotis* provided by Fibiger (1990), Yela (1992), Fibiger et al. (2010), Garretas et al. (2018) and Garretas and Blázquez (2023). Dissections were performed using a standard procedure (Yela 1992; Fibiger 1997) with minor modifications. Images of the adults (Figs 1–3) were taken with a Canon EOS 750D digital camera, were z-stacked using the software Zerene (Zerene Systems, Richland, Washington, USA) and were edited with Thumbplus 10. Morphology of male (Figs 5A, B) and female (Fig. 6) genitalia were studied using a Motic SMZ-171 (Hong Kong, China) stereomicroscope with a Moticam Pro 282A digital camera. Microphotographs of the genitalia were taken, measured, and edited with Motic Images Plus v. 2.0. The abbreviation “g. prep.” refers to the number of a genitalia preparation.

### ﻿Molecular study

Legs of four adult specimens of *Agrotisvillenensis* (Table 1) were processed and sequenced at the Canadian Centre for DNA Barcoding (CCDB, Guelph, Ontario, Canada) to obtain DNA barcodes using the standard high-throughput protocol described by deWaard et al. (2008) (https://www.dnabarcoding.ca/pa/ge/research/protocols). No publicly available sequences from Spain were available from BOLD prior to this study. Ultimately, the analysis included 322 *Agrotis* COI sequences (with > 500 of 658 base pairs) from Europe including public sequences downloaded from Public Data Portal of BOLD. Voucher data, GPS coordinates, images, sequences, Genbank Accession, and trace files are publicly available through the public data set (https://doi.org/10.5883/DS-VILLENEN) in BOLD. Sequences were compared to a reference library of Lepidoptera barcodes using the identification engine (BOLD-ID).

**Table 1.** Interspecific mean K2P (Kimura 2-Parameter) divergences (mean pairwise distances) based on the analysis of COI fragments (>500 bp) among *Agrotisvillenensis* and other *Agrotis* and outgroup species (GRA: *A.graslini*; VES: *A.vestigialis*; VIL: *A.villenensis*; SIM: *A.simplonia*; SPI: *A.spinifera*; SEG: *A.segetum*; REC: *E.recussa*).

	GRA	VES	VIL	SIM	SPI	SEG	REC
*A.graslini*		3.34	3.50	6.08	5.17	6.23	8.28
*A.vestigialis*			1.37	3.80	4.56	5.02	5.78
*A.villenensis*				4.10	5.02	5.02	6.08
*A.simplonia*					4.26	4.56	5.62
*A.spinifera*						4.71	5.62
*A.segetum*							6.69

**Figure 4.**
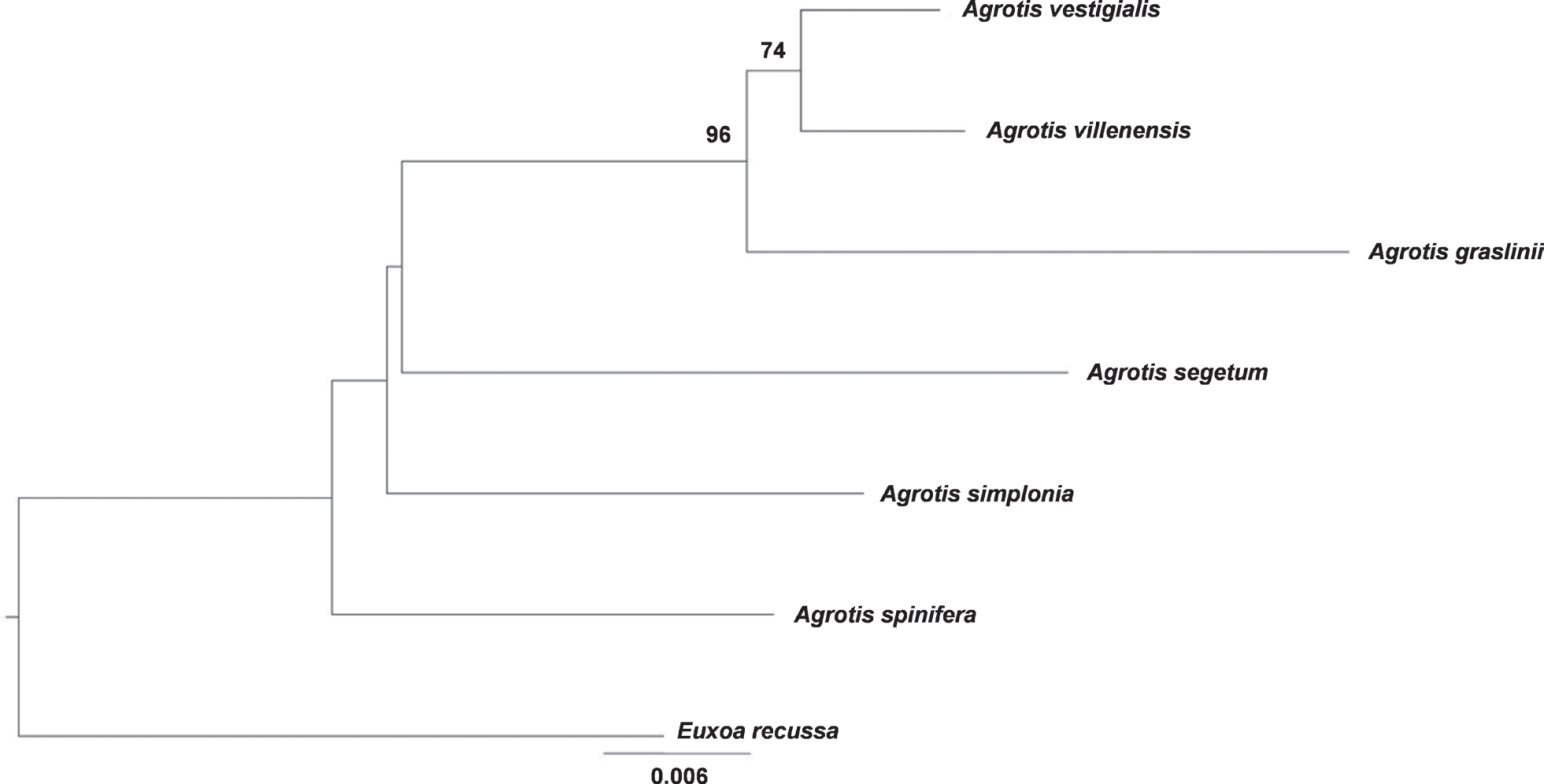
Maximum-likelihood tree of *Agrotis* species, obtained from 322 nucleotide COI sequences. The depth of each branch shows divergence within lineages. Bootstrap values are provided at major nodes. The scale bar represents 0.006 genetic difference.

Sequence divergences of barcodes were calculated using the Kimura 2-parameter (K2P) model (Kimura 1980) and interspecific genetic distances were calculated using the analytical tools of BOLD. All the new and publicly available barcode sequences were downloaded and aligned with the CLUSTAL algorithm of the MEGA6 software (Tamura et al. 2013). Bootstrap values were calculated with 1000 replicates, and initial neighbor-joining (NJ) and maximum likelihood (ML) trees based on distance were constructed with the MEGA6 software. We selected the Iberian *Agrotis* nominal species with the same Barcode Index Number as *Agrotisvestigialis* (BOLD:AAD1898; *n* = 57) according to Yela et al. (2011), including sequences from initially identified as *Agrotischaroae* Yela, Fibiger, Zilli & Ronkay, 2010, *Agrotisgarretasorum* Blázquez, Garretas & Gaytán, 2018, *Agrotissabulosa* Rambur, 1837 and *Agrotisyelai* Fibiger, 1990 (Fig. 3), and *Agrotisgraslini* Rambur, 1848 (BOLD:AAZ5040; *n* = 4), *Agrotissimplonia* (Geyer, 1832) (BOLD:ABZ7031; *n* = 22), *Agrotisspinifera* (Hübner, 1808) (BOLD:AAE4276; *n* = 3) and *Agrotissegetum* ([Denis & Schiffermüller], 1775) (BOLD:AAC3848; *n* = 236) for congeneric comparison, and *Euxoarecussa* (Hübner, 1817) which is taxonomically related as outgroup to root the tree. To assess the COI divergences between the taxa, we included all sites with the pairwise deletion option. All trees produced identical topologies; therefore, only the ML tree is presented (Fig. 4).

### ﻿Repository abbreviations

**DZPA** UM: Research Collection of Animal Biology at the Department of Zoology and Physical Anthropology of the University of Murcia, Spain.

**JLY** UCLM: Research Collection of José Luis Yela, University of Castilla-La Mancha, Toledo, Spain.

**MNCN**National Museum of Natural Sciences, Higher Council for Scientific Research (CSIC), Madrid, Spain.

## ﻿Results

### ﻿ Agrotis villenensis sp. nov.

Taxon classificationAnimaliaLepidopteraNoctuidae

6ED46B4A-B3F5-5C3E-8EE7-5B7CDDE63104

https://zoobank.org/3FF9F1CD-FF9A-4D17-9BD9-E9C678582491

#### Type material.

***Holotype***, male. SPAIN • province of Alicante: Villena, Peña Rubia; 38.6092, -0.8043; 620 m elev.; 23 Sep 2023; g. prep. JLY90.1892; leg. J. L. Yela & D. Molina, in coll. JLY, UCLM (catalogue number T-JLY-039). ***Paratypes*.** 2 males, 4 females; same locality as holotype • 13 Sep 2010; leg. D. Molina (1 male), in coll. JLY, UCLM (catalogue number T-JLY-040) • 23 Sep 2023; g. prep. JLY90.1981, IBLAO3089-24; leg. J. L. Yela & D. Molina (1 female), in coll. MNCN (catalogue number T-JLY-041) • 23 Sep 2023; g. prep. JLY90.1890, IBLAO3090-24; leg. J. L. Yela & D. Molina (1 female), in coll. JLY, UCLM (catalogue number T-JLY-042) • l9 Sep 2020; g. prep. JLY90.1889, IBLAO3037-23; leg. D. Molina (1 male), in coll. JLY UCLM (catalogue number T-JLY-043) • 23 Sep 2023; leg. J. L. Yela & D. Molina (1 female), in coll. MNCN (catalogue number T-JLY-044) • 28 Sep 2011; IBLAO2068-21; leg. D. Molina (1 female), in coll. DZPA, UM (catalogue number T-JLY-045).

#### Additional material.

Four additional specimens were recorded as photographs, all from the same locality: one male, 15 Sep 2019, and 3 females, 26 Sep 2009, 13 Sep 2010 and 17 Sep 2020.

#### Diagnosis.

Externally, very similar to *A.vestigialis* (Figs 1–3), itself a very variable species (e.g., Skinner 1984; Fibiger 1990). The ground color of the forewings of *A.villenensis* generally are pale greyish, with a brownish or pinkish tinge. Cross lines are more diffuse than in most *A.vestigialis*, so that some specimens resemble *A.sabulosa*. Hind wings are mostly purely white, although some females show a light greyish terminal area. Male antennae are bipectinate, except in the apical antennomeres. The number of non-pectinate apical antennomeres is 13 in the three males studied, whereas the modal number in *A.vestigialis* is 15 (*n* = 16). The number of non-pectinate antennomeres is fewer in the other members of the *Agrotisvestigialis* species group.

**Figure 5.**
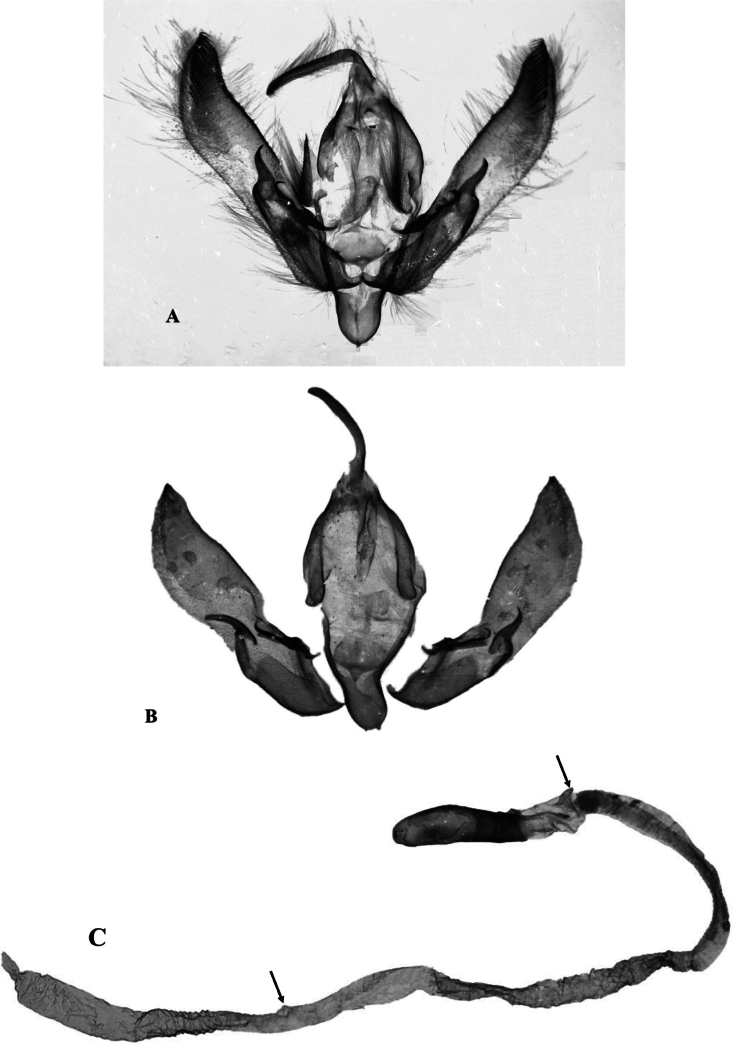
Male genitalia of *A.villenensis* sp. nov. **A** external capsule of the holotype (g. prep. JLY90.1892) **B** external capsule of a male paratype, pressed to better show the curved shape of the valvae (g. prep. JLY90.1891) **C** phallus with everted vesica; arrows indicate the digitiform, subbasal diverticulum and the median diverticulum (g. prep. JLY90.1892). Photographs: Mateo Yela Berzosa.

The male genitalia of *A.villenensis* are similar to those of the related species in the *A.vestigialis* species group, but with four key distinctive features (Fig. 5A–C). First, the valvae are broader than in other species, and the apical cuculli are more sharply pointed. Second, number of spiniform setae comprising the corona is 16–17 in *A.villenensis* and 20–24 in *A.vestigialis* (*n* = 8). Third, the basal swelling in the vesica of *A.villenensis* is similar to that of *A.vestigialis*, but the subbasal diverticulum is narrower and situated dorsolaterally, ventrolaterally in *A.vestigialis*. And fourth, the vesica of *A.villenensis* is very long and has a small median diverticulum, not present in any other European *Agrotis*, and a very broad apical swelling, corresponding to the broad distal end of the appendix bursae of the female genitalia (for comparison, see Fibiger 1997). The vesica is partially collapsed in Fig. 5C, so that the apical swelling appears smaller than it is when the vesica is fully inflated.

**Figure 6.**
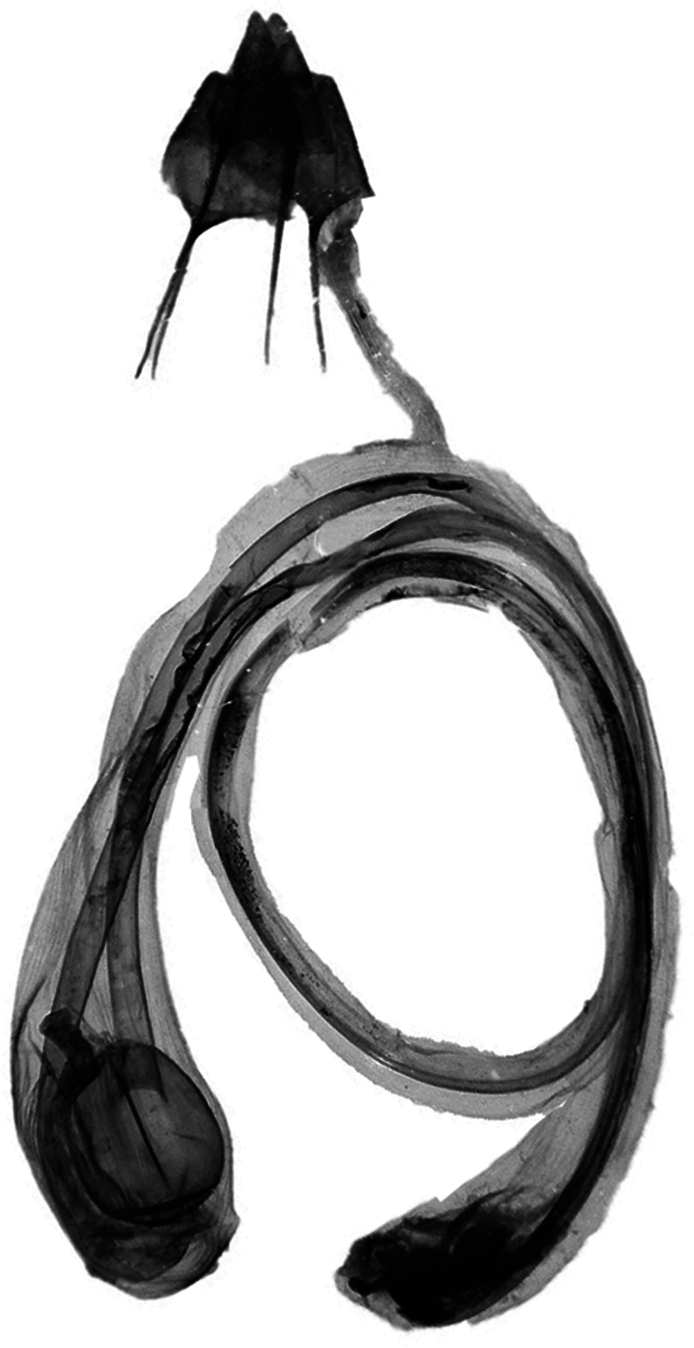
Female genitalia of *A.villenensis* sp. nov. (g. prep. JLY90.1890). Photograph: Mateo Yela Berzosa.

The female genitalia of *A.villenensis* (Fig. 6) are very similar to those of *A.vestigialis*, from which it can be distinguished by three key features: first, the ovipositor is considerably shorter; the appendix bursae is longer, although as in *A.vestigialis* it only loops once; and the distal part of the appendix bursae is relatively large and globose.

The four known COI sequences of *A.villenensis* are identical and form BIN BOLD:AEM2348 (sequence length 658 bp). This new species differs from *A.vestigialis* by at least 1.37% (BOLD:AAD1898; n = 55; mean 1.04; maximum distance 2.73%) (Table 1).

#### Description.

Wingspan: males 31.6–40.0 mm (mean 35.7; *n* = 3), females 33.9–40.0 mm (mean 36.7 mm, *n* = 4). Groundcolor of the forewings greyish, in the males with brownish tinge, in the females pinkish. ***Head***: male antennal segments bipectinate and ciliate, as in *A.vestigialis*; 13 distal antennomeres ciliate. Female antenna filiform. Labial palpi short. Haustellum long, well developed. ***Thorax***: dark greyish patagia; tegulae light gray, with dark anterior border. ***Wings***: design of the forewings generally low contrast. Antemedial (basal) line inconspicuous, as a double strip delimiting the clearly visible ochreous basal spot towards its external side and, in some specimens, forming a grayish basal wedge that extends beyond the basal spot. Postmedial line usually less marked or even absent (Fig. 2A), but sometimes well marked, wavy (Fig. 2D). Subterminal line almost absent; 4–6 short saggital stripes between veins R3–R4 and Cu1–Cu2. Terminal line as a row of tiny intervenal 7 or 8 dots. Claviform spot dark, elongate. Orbicular spot small, usually elongate, filled with a light-grey ellipse with a dark spot in its centre. Reniform spot large, dark grey, with a thin clear edge inwards. Hindwings pure white in males, white with greyish suffusion towards the termen in females, with greyish discal spot. ***Abdomen***: very small dorsal tufts in segments A2 to A4 in males; absent in females. ***Male genitalia***: uncus slender, relatively long, pointed ventrad. Valval costa with a small distal hump before the sharply pointed cucullus; valva elongate, but relatively wide. Corona as a row of 16 or 17 spines. Harpa, tegumen, fultura and vinculum as in the rest of the *Agrotis* species. Phallus short. Vesica very long, with a smooth basal swelling, a narrow dorso-lateral subbasal diverticulum and a tiny median diverticulum. ***Female genitalia***: ovipositor short, conical, and peaked. Appendix bursae very long, looping once; its distal part is relatively large and globose.

#### Distribution.

**Figure 7.**
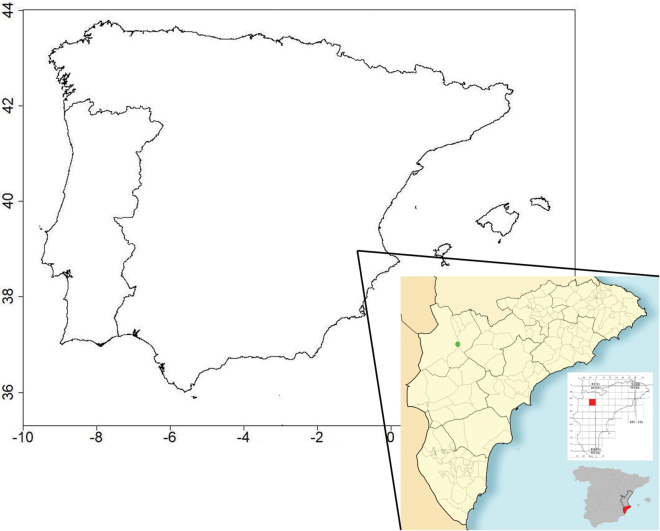
Location of Peña Rubia, Villena, the type locality of *Agrotisvillenensis* sp. nov., in the province of Alicante and the Iberian area.

*Agrotisvillenensis* appears to be endemic to the southeastern Iberian Peninsula. It is only known from one locality in the inland northern Alicante province (Fig. 7).

#### Biology and habitat.

The adult is active in September in one apparently short generation. The early stages are unknown. All known specimens were collected in a hilly area at 620 m elevation, just a few metres from a forest area dominated by pine forests of *Pinushalepensis* Mill. and associated scrub. The soil is sandy due to an inland dune of eolian origin, consisting of sand-sized quartz and limestone particles. This sandy area occupies a strip of land about 8 km long and between 300 m and 2 km wide, which runs from the shadow of the Peña Rubia mountain range, continuing along the slopes of the Frare mountain range between the municipalities of Villena and Biar. The area also contains crops in production (mainly olive and almond trees) and country houses. The sabulicolous vegetation extends along hills, ravines, fields without tillage and crop margins, distinguished by the presence of stone pine (*Pinuspinea* L.) and various other plants as *Maresianana* (D.C.) Batt, *Teucriumdunense* Sennen, SideritischamaedryfoliaCav.subsp.chamaedryfolia and LinariadepauperataLeresche ex Langesubsp.hegelmaieri (Lange) De la Torre, Alcaraz & M.B. Crespo.

#### Etymology.

The name of the species is dedicated to the city of Villena in the province of Alicante, where all known specimens were collected.

## ﻿Discussion

In *Agrotis*, some species groups can be relatively easily recognized, such as the *A.fatidica* Hübner, 1824 species group, which was studied by Ronkay and Huemer (2018), or the *A.vestigialis* group. Prior to 1990, the Iberian Peninsula *Agrotisvestigialis* species-group included the nominate species and *A.sabulosa* Rambur, 1839 (Agenjo 1947, 1977; Calle 1983). Subsequently, three additional species were added: *A.yelai* Fibiger, 1990, *A.charoae* Yela, Fibiger, Zilli & Ronkay, 2010, and *A.garretasorum* Garretas, Blázquez & Gaytán, 2018. Although it is beyond the scope of the present article, the taxonomic relationships of all these species should be revisited; all share the same BIN with *A.vestigialis*, according to material available at BOLD (https://boldsystems.org/; Ratnasingham and Hebert 2007).

In several groups of invertebrate taxa, differences in barcode sequences higher than 2% are typical of interspecific variation (Hausmann et al. 2011) and recognized as distinct Molecular Taxonomic Units (MOTU), while lower values often correspond to intraspecific differences. However, the divergence between young sister species may fall below the 2% threshold, while unusually variable species may exceed it. This is an immediate consequence of the gradual process of speciation, and nominal species do not always correspond to the same divergence stage (e.g., de Queiroz, 2007). *Agrotisvillenensis* differs from the other Iberian *Agrotis* species that have been compared in this paper by an average of 3.80% (range 1.37–5.02%) (Table 1) and belongs to a phylogenetically isolated lineage that is well supported by morphology and genetic data (Fig. 4). These barcode differences are larger than those of some other recently recognized *Agrotis* species in other species groups (e.g., *A.mazeli* Ronkay & Huemer, 2018 and *A.mayrorum* Ronkay & Huemer, 2018 by 1.12%, or *A.proverai* Zilli, Fibiger, Ronkay & Yela, 2010 and *A.fatidica* by 0.5%).

The wing pattern of *A.villenensis* is similar to that of the very variable *A.vestigialis*, whilst male and female genitalia are also similar but with distinctive features, as explained in the diagnosis of the new species above. The small number of specimens studied hinders a statistical analysis of these features, although genitalia and barcode differences and geographic isolation support the species status of *A.villenensis*.

Members of the *A.vestigialis* species group are associated with herbaceous plants growing on sandy soils, where their larvae feed on the roots of several herbaceous plants (Bergmann 1954; Skinner 1984; Fibiger 1990). The sandy area around Peña Rubia extends between the municipalities of Villena and Biar and farther east. There are several other sandy patches in the surrounding area that may harbor populations of this species, which should be investigated. The nearest known populations of *A.vestigialis* are in the province of Tarragona, in Catalonia (J. L. Yela, pers. obs.), about 350 km north of Villena. It will be interesting to locate intermediate populations in suitable habitats and to confirm their identity to establish the distribution limits of both species.

The fact that *A.villenensis* has remained unknown to science so far precludes informed inferences about the degree of threat to which the species may be submitted to. For this reason, it would be important to intensify sampling in supposedly suitable places, in principle not strictly coastal sandy areas located approximately between the Spanish provinces of Murcia and Tarragona.

## Supplementary Material

XML Treatment for Agrotis villenensis

## ﻿Additional information

### Author ORCIDs

José Luis Yela https://orcid.org/0000-0003-1371-8495

David Molina https://orcid.org/0000-0002-6057-831X

Antonio S. Ortiz https://orcid.org/0000-0002-3877-6096

### Data availability

All of the data that support the findings of this study are available in the main text.
